# Prevalence of Mycotoxins and Endotoxins in Total Mixed Rations and Different Types of Ensiled Forages for Dairy Cows in Lithuania

**DOI:** 10.3390/toxins13120890

**Published:** 2021-12-12

**Authors:** Gintarė Vaičiulienė, Bronius Bakutis, Jurgita Jovaišienė, Rimvydas Falkauskas, Gediminas Gerulis, Sigita Kerzienė, Violeta Baliukonienė

**Affiliations:** 1 Department of Food Safety and Quality, Faculty of Veterinary, Lithuanian University of Health Sciences, Tilzes Str. 18, LT-47181 Kaunas, Lithuania; bronius.bakutis@lsmuni.lt (B.B.); jurgita.jovaisiene@lsmuni.lt (J.J.); rimvydas.falkauskas@lsmuni.lt (R.F.); gediminas.gerulis@lsmuni.lt (G.G.); violeta.baliukoniene@lsmuni.lt (V.B.); 2 Department of Animal Breeding, Faculty of Animal Science, Lithuanian University of Health Sciences, Tilzes Str. 18, LT-47181 Kaunas, Lithuania; sigita.kerziene@lsmuni.lt

**Keywords:** endotoxins, haylage, mycotoxins, silage, total mixed ration

## Abstract

In this study, 119 samples of total mixed rations and different types of ensiled forage (maize and grass silage, and haylage) collected in 2019–2020 from dairy farms in Lithuania were analyzed to evaluate the quantitative occurrence of mycotoxins and endotoxins. Samples were analyzed using high-performance liquid chromatography (HPLC) with a fluorescent (FLD) and an ultraviolet detector (UV) of mycotoxins and a detection assay based on the ELISA technology for endotoxins. The study included toxins regulated within the European Union (aflatoxin B1 (AFB1), zearalenone (ZEA), deoxynivalenol (DON) and T-2 toxin) and nonregulated toxins (endotoxins). Mycotoxin analysis showed that 49.58% of the samples out of 119 were positive for AFB1, 52.11% for ZEA and DON, 55.47% for T-2 toxin and 84.04% for endotoxins. In the contaminated samples, the highest mean values of AFB1 and T-2 toxin were determined in the grass silage samples, while ZEA and DON–were determined in the maize silage samples. Maize silage samples had the highest ZEA and DON concentrations, exceeding the EU maximum permissible concentration limits. In the haylage samples, AFB1 mycotoxin exceeded the maximum concentration limits. The highest mean value of endotoxins was determined in the total mixed rations samples. This is the first study to provide information about the concentrations of mycotoxins and endotoxins in total mixed rations and different types of ensiled forages for dairy cows in Lithuania.

## 1. Introduction

Ensiling is a method of forage conservation. It is based on the natural fermentation of lactic acid under anaerobic conditions. Nowadays, silage and total mixed rations are the main source of feed in the diet for dairy cows in many parts of the world. The most commonly used for forage ensiling are legumes, haylage, grasses and maize. There are several factors that can affect the hygienic quality of silage such as improper silage preparation, compression and moisture content, mold and bacteria growth and the production of mycotoxins and endotoxins [1].

The pH of ensiled forage is a measure of the acidity and the quality of the silage. Dairy cows are fed staple forage, mainly whole plants, with a pH between 5.5 and 6 immediately after grinding. During ensiling, the lactic acid, produced by lactic acid bacteria (LAB), occurs in the highest concentrations in the silage and is the main contributor to the reduction of the pH concentrations during fermentation, as it is approximately by 10 to 12 times stronger than any other major acid, like acetic acid or propionic acid found in silages. Under normal feeding conditions, the lactic acid from the silage is converted to propionic acid in the rumen. 

The final pH of the silage is influenced by many different factors, but it is mainly related to the lactic acid concentration and the buffering capacity of the raw material. Respectively, maize silages have a lower final pH (3.7–4.0) than grass silages (4.3–5.0) because they have lower buffering capacities. Thus, the pH values of the different compositions of ensiled forage determine the silage fermentation and hygienic quality [2].

Mycotoxins are low molecular weight toxic secondary metabolites produced by several species of filamentous fungi, such as *Aspergillus* spp., *Fusarium* spp., *Penicillium* spp. or *Alternaria* spp., and they are very often found in the feedstuffs for dairy cows. These toxic compounds can occur before, during or after harvest. AFB1, DON, ZEA and T-2 toxin are the main mycotoxins found in silages [1]. The European Union regulation on feedstuffs has so far established aflatoxins (AFB1, AFB2, AFG1, AFG2) by Directive 32/2002 (European Communities 2002). Moreover, additional “guidance values” have been published by the European Commission for several other compounds such as DON, ZEN, fumonisins (FB1, FB2), ochratoxin A (OTA) (European Commission 2006) and for T-2 and HT-2 toxins (European Commission 2013) [3]. The guidance values for AFB1, ZEN, DON and T-2 toxin in the EU are shown in Table 1.

Feed and food contaminated by mycotoxins can have a significant adverse impact on animal health and productivity and may also cause health problems in humans. Feed contaminated with these toxins can cause mycotoxicosis in animals, characterized by a variety of clinical signs depending on the toxin, which can cause significant losses to the ruminant industry [4]. Mycotoxins in cattle usually not only affect the nutrient uptake and digestion, but also the intestinal histomorphology, intestinal barrier integrity, mucin production, microbial composition and the local immune system. Moreover, they can be harmful to animal health due to various modes of action, causing immunomodulatory, neurotoxic, hepatotoxic, nephrotoxic, and genotoxic effects, as well as developmental and reproductive disorders [5].

*Enterobacteriaceae* are facultative anaerobic Gram-negative bacteria found in poorly preserved silage [6]. Their metabolism varies depending on the different species: lactic, acetic and succinic acids, 2,3-butanediol, ethanol, CO_2_ and water can be produced. Some species of the *Enterobacteriaceae* group can decompose proteins to produce ammonia and biogenic amines that are undesirable in silage [7]. During silage fermentation, the raw material is not sterilized, so the microbiota initiating the fermentation consists of the natural microbiota of the forage plant. Despite the great diversity of silage microorganisms, some groups have been identified more frequently and the bacteria of the *Enterobacteriaceae* family, spore-forming bacteria (*Bacillus* and *Clostridium*), *Listeria* and acetic acid bacteria are considered undesirable during ensiling [8]. Gram-negative bacteria have endotoxins on their outer membrane which are released during cell lysis [9]. Animal health conditions are associated with sensitivity to these compounds; however, ruminants can adapt to the presence of these toxins in their diet. Most of the microorganisms from this group found in silage are not pathogenic, but some species or strains may be pathogenic [8]. Epiphytic *enterobacteria*, such as *Erwinia herbicola* and *Rahnella aquitilis*, often dominate in fresh crops and grass, but after ensiling these species are rapidly superseded by *Hafnia alvei*, *Serratia fonticola* and finally by *Escherichia coli*. *Serratia fonticola* usually may appear early on if the pH of the silage falls very rapidly. *E. coli* is the most important species in this group from the viewpoint of the risk to health [10]. *E. coli* O157:H7 *Shiga*, a toxin-producing (STEC) strain, can be found in silage and also in cattle, which are considered to be the main reservoir of this strain [8]. Yet, according to the results of previous studies, after the experimental inoculation in alfalfa, it does not survive after 100 days of ensiling [11].

Endotoxins are lipopolysaccharides (LPS) found in all Gram-negative bacteria cell wall outer membranes which can cause septic shock in animals. LPS has a molecular weight which is >100,000 Daltons. LPS consists of two main components, hydrophilic lipid A and hydrophilic polysaccharide (O-region), which are important for the biological activity of endotoxins. Their toxicity is related to the lipid component and the immunogenicity to the polysaccharide components [12]. Endotoxins can be extremely toxic to dairy cows at parenteral doses of 100 ng/kg body weight. Through the feed, water, air, dust, feces and all of the environment, cattle are constantly in contact with endotoxins produced by Gram-negative bacteria. After entering the blood system, endotoxins may cause endotoxicosis. The early signs of moderate-to-severe endotoxicosis can be salivation, urination or defecation. Moreover, only very high doses of endotoxins may be a significant factor in the pathogenesis of a variety of clinical conditions, from ruminal acidosis, laminitis, Gram-negative infections through to shock and ‘sudden death syndrome’ in dairy cattle [13].

The hypothesis of this study was that in total mixed rations and different types of ensiled forage may be found the toxic secondary metabolites of fungi and the outer membrane components of Gram-negative bacteria. The aim of this study was to evaluate the quantitative prevalence of mycotoxins and endotoxins in total mixed rations and different types of ensiled forages for dairy cows in Lithuania.

## 2. Results and Discussion

### 2.1. Analysis of pH Values

In this work, the pH of total mixed rations and the different types of ensiled forages were measured in order to evaluate whether the investigated samples met the good quality feed pH requirements. The results of the analysis and the mean values of the pH of the total mixed rations and the different types of ensiled forages are summarized in Table 2.

The highest mean values of the pH were determined in the TMR samples, the medium and similar values in the grass silage and the haylage samples, while the lowest were in maize silage samples. Variation of the pH values in the TMR samples may be caused by the different composition of the samples. The grass silage, haylage and the TMR samples presented higher values of pH than the maize silage samples. In general, the mean pH values of the different samples of the TMR and the ensiled forage were within the range of values suggested for the different types of well-preserved stage [14].

### 2.2. Analysis of Total Count of Enterobacteriaceae

The results of the total count of *Enterobacteriaceae* in the TMR and the different types of ensiled forage in Lithuania are shown in Table 3.

The total count of *Enterobacteriaceae* in the investigated samples varied from 3.00 to 4.80 log_10_ CFU/g. The highest mean value of the total count of *Enterobacteriaceae* was determined in the maize silage, while the lowest was in the haylage in a different number of samples. The count of *Enterobacteriaceae* is 22.14% higher in the maize silage than in the haylage samples. Previous studies have demonstrated the effect of endotoxins, found in feed, on Gram-negative bacteria [15]. Therefore, for this reason, in this work we also investigated the count of *Enterobacteriaceae* in the samples of total mixed rations and different types of ensiled forages.

### 2.3. Analysis of Mycotoxins

In this section of the mycotoxins study, the total set of collected samples was analyzed. The maize silage and the TMR samples were the most contaminated with AFB1, ZEA, DON and T-2 toxin mycotoxins. This could be explained by their different compositions: usually maize crops contain more proteins and polysaccharides that can help fungi, bacteria and other plant pathogens to grow and survive. The mean value of the contaminated samples was calculated by taking into account that the toxin concentration in the negative samples was equal to the detection limit. The results of the analysis are summarized in Table 4.

Fifty-nine (49.58%) samples out of 119 were positive for AFB1. The highest mean value of AFB1 in the contaminated samples was determined in the grass silage samples (six positive samples), while the lowest mean value was in the TMR (31 positive samples) samples. Moreover, the mean values of contamination have always been below the EU guidance values. Despite the low level of occurrence, the TMR was the most AFB1 contaminated type of feed. It has been determined that AFB1 frequency and levels are relatively low compared to other mycotoxins in well-preserved silos [16]. Based on the results of other research, in many European countries AFB1 mycotoxins are not considered a major problem and has been rarely reported, whereas their presence is mostly associated with geographical regions [3]. In addition, previous studies indicate the absence of AFB1 in feed samples [17,18]. On the other hand, in agreement with our results, other studies have also detected AFB1 in a small number of feed samples and at low concentrations [19,20]. Moreover, in some studies, AFB1 have been detected at much higher levels in maize silage [21].

Analysis of the fusarium toxins showed that sixty-two samples out of 119 (52.12%) for ZEA and DON and sixty-six samples (55.47%) for the T-2 toxin were positive. Among the feed of different botanical compositions, fusarium mycotoxins were detected in the maize and grass silage, the haylage and the TMR. The mean values of the mycotoxins detected in the silage positive samples did not exceed the EU guidance values. The highest mean value of ZEA and DON in the contaminated samples were determined in the maize silage samples (22 positive samples for ZEA and 14 for DON), while the lowest amount of ZEA was determined in the grass silage samples (six positive samples) and DON in the haylage samples (eight positive samples). The results of the present study showed that the prevalence of fusarium mycotoxins is relatively low in the haylage samples. Meanwhile, the maize silage and the TMR samples were contaminated more frequently than the other ensiled forage. Similar to our results, DON has been reported to be the most commonly found mycotoxin in silage and may be found in higher concentrations [22]. Earlier findings [22] reported the mean values of DON similar to what was detected in the current publication, at a maximum concentration of 750 ug/kg. Moreover, in Poland, Kosicki et al. [22] and Panasiuk et al. [3] detected high levels of DON with a mean value of 633 and 447 μg/kg, respectively. In Ireland, Cogan et al. [23] detected DON in maize silages in an average concentration of 603 μg/kg and 711 μg/kg as the maximum concentration. ZEA has also been reported as usually occurring in ensiled feed. This toxin, as is also confirmed by our results, has been detected in higher concentrations than DON. Higher mean values were also reported by Driehuis et al. [24] and Cogan et al. [23] in concentrations of 174 and 209 μg/kg, respectively. T-2 toxin in our investigated ensiled feed samples occurred rarely and in low concentrations. The highest mean values of T-2 toxin (154.17 μg/kg) were detected in the grass silage, and the lowest in the haylage, at 96.15 μg/kg in the samples. The T-2 toxin concentrations were 1.6 times higher in the grass silage samples than in the haylage samples. The T-2 toxin has been detected in grass and maize silages at a mean value of 154.17 ug/kg and 110.09 ug/kg, respectively. Moreover, Venclovas et al. [25] found similar T-2 concentrations in silage samples from Lithuania, in concentrations of 104 μg/kg and 199 μg/kg, respectively. Moreover, in some studies and countries, considerably lower levels of T-2 toxin have been detected in maize and grass silages [3].

### 2.4. Analysis of Endotoxins

The results of the endotoxin concentrations in the total set of the analyzed samples are shown in Table 5.

One hundred (84.04%) samples out of 119 were positive for endotoxins. The highest mean values of endotoxin concentrations were determined in the TMR samples, while the lowest were in the grass silage samples. Endotoxin concentrations are 2.36 times higher in TMR samples than in grass silage samples. In comparison with the TMR samples, lower mean values of endotoxin concentrations were found in the maize and haylage samples at 9.15% and 57.8%, respectively. There is a serious lack of information on the prevalence of endotoxins in cattle feed, especially in various types of ensiled feed. Through feed, water and environment, cattle are in constant contact with endotoxins. Therefore, endotoxin exposure is most important in agriculture and related farming industries, in cereal, cotton, linen and vegetable processing industries and also in the animal feed industry. Lipopolysaccharides can be analyzed to obtain information on the hygienic quality of the feed, and LPS shows the overall effect of the feed on Gram-negative bacteria [26]. Based on previous publications, we can assume that many animal feeds may be contaminated with endotoxins. Moreover, Wichert et al. [27] has found a very high content of endotoxins in straw and roughage feed samples. Farm livestock are constantly exposed to endotoxins in their feed and environment, and also to large quantities of endotoxins present in Gram-negative bacteria in the gastrointestinal tract. Cort et al. [28] reported concentrations of 12.4 and 12.9 ng endotoxin/mg in pig feed. The same authors measured 0.05 ng/mg of endotoxins in haylage and 10 ng/mg in granulated goat feed. Dutkiewicz et al. [15] reported that the maize silage samples from the center of the silo contained one EU endotoxin/mg but 200 × higher concentrations were found in the samples from the surface. Moreover, Wallace et al. [29] concluded that animal feed may be contaminated with endotoxins on a regular basis, with concentrations of endotoxins of 1000 EU/mg in feed not being unusual. Accordingly, based on the results of our research, where in different types of ensiled forage samples the endotoxin concentrations were determined from 23.49 to 678.13 EU/mL, we can assume that these small quantities of endotoxins may not cause clinical signs and may be harmful to cattle. This assumption is based on the fact that ruminants already have high levels of endotoxins in the ruminal digestive, and they are more resistant to endotoxins contaminating feed. The exception would include cattle, whose gastrointestinal disorders have disrupted the barrier function of intestinal tissues. For these animals, feed ingredients containing endotoxins are not recommended [29].

## 3. Conclusions

The results of our study imply that total mixed rations and different types of ensiled forage in Lithuania may be a potential source of mycotoxins and/or endotoxins. Forage produced in Lithuania during the period of this study was mostly contaminated with DON and ZEA mycotoxins, though in small quantities. The highest concentrations of DON and ZEA were determined in maize silage samples, while AFB1 and T-2 toxin were in grass silage samples. Maize silage was found to be more sensitive to mycotoxin contamination than grass silage, haylage and TMR. In addition, high levels of mycotoxins were found in different types of ensiled forage, and the effects of their mixture may cause chronic problems in exposed cattle, which may have synergistic and/or side effects. In the investigated samples of different types of ensiled forages, endotoxin concentrations varied from 23.49 to 678.13 EU/mL. The highest mean values of endotoxins were determined in the TMR samples, while the lowest were in the grass silage samples. Moreover, further research is necessary for the evaluation of possible adverse effects on cattle health and productivity produced by certain concentrations of mycotoxins and endotoxins in total mixed rations and ensiled forage.

## 4. Materials and Methods

### 4.1. Samples

One hundred and nineteen mold-free samples of different types of ensiled feed consisting of maize silage (*n* = 34), grass silage (*n* = 20), haylage (*n* = 14) and total mixed rations (TMR) (*n* = 51) were collected from 41 conventional dairy farms of Lithuania. Samples were collected between September 2019 and July 2020. The sampling locations were spread throughout the country: in its northern, southern, central, western and eastern parts. The feed samples, each weighing about 5 kg each, were transported immediately to the laboratory in polyethylene bags with a minimum air level. The samples were stored in the dark at −20 °C until the date of the analysis.

### 4.2. Determination of the pH Value

The pH level was measured by electrometric method in diluted feed samples with a pH meter (WTW^®^inoLab pH 720, Weilheim in Oberbayern, Germany) fitted with a glass electrode. A 25 g feed sample was homogenized with 100 mL distilled water for 25–30 min, then the mixture was passed through the filter paper, and after 15–20 min the pH value was measured in three repetitions. After setting a constant value, the pH was determined from the dial reading with an accuracy of at least 0.05 pH unit.

### 4.3. Determination of the Total Count of Enterobacteriaceae

The total count of *Enterobacteriaceae* in total mixed rations and different types of ensiled feed were defined using “Microbiology of the food chain–Horizontal method for the detection and enumeration of *Enterobacteriaceae*–Part 2: Colony-count” technique (ISO 21528-2:2017, corrected version 2018-06-01). Feed samples weighing 10 g each were homogenized with 90 mL of buffered peptone water (BPW) for 20 min. Then 1 mL of the homogenized samples were transferred into a Petri dish and poured with approximately 15 mL of violet red bile glucose (VRBG) agar medium. Samples with the medium were carefully mixed by horizontal movements and the medium was allowed to solidify with the Petri dishes standing on a cool surface. After complete solidification of the medium, a covering layer of approximately 5 to 10 mL of the VRBG agar medium was added to prevent spreading growth and to achieve semianaerobic conditions. The prepared Petri dishes were inverted and incubated at 37 °C for 24 h ± 2 h. The total count of *Enterobacteriaceae* in feed samples were determined by counting the characteristic colonies: pink to red or purple (with or without precipitation haloes).

### 4.4. Determination of Mycotoxins Concentrations by HPLC

Contamination with AFB1, ZEA and T-2 toxin in total mixed rations and different types of ensiled forages was tested by high-performance liquid chromatography (HPLC) with a fluorescent detector (FLD) (Model LCMS-8060 Shimadzu Corp., Kyoto, Japan) and DON by HPLC with mass spectrometry and an ultraviolet detector (UV) (Model Sciex API 5000, McKinley Scientific, NJ, USA). The samples were air-dried, ground to pass through a 1 mm sieve and homogenized. Extraction of samples was performed in distilled water for DON, in methanol:water (75:25 *v*/*v*) for AFB1 and ZEA and in methanol:water (60:40 *v*/*v*) for T-2 toxin under stirring on a mechanical shaker (Phoenix Instrument RS-OS 20, Inc., Garbsen, Germany) for 60 min at 23 °C. After extraction, samples were centrifuged at relative centrifugal force (RCF) of 3468× *g* for 10 min (Centrifuge MPW-251, MPW, Warsaw, Poland). Subsequently, the supernatants were filtered with PTFE syringe filters through 0.22-μm diameter pores (Millex-GS, Millipore, Billerica, MA, USA) and diluted with phosphate buffered saline (PBS). Then, for the sample clean-up step, extracts were passed over a multi-mycotoxin immunoaffinity column 11+Myco MS-PREP^®^ (R-Biopharm AG, Pfungstadt, Germany) according to the manufacturer’s recommendations and diluted with distilled water. The prepared samples were analyzed by high-performance liquid chromatography with the parameters which are presented in Table 6. Chromatographic mycotoxin separation was achieved using a LiChrospher^®^ 100 RP-18, LiChroCART 250-4 column (250 × 4.0 mm, 5 μm; Supelco Park, Bellefonte, PA, USA). Mycotoxins were determined by comparing peak retention times with standard solutions. The concentrations of mycotoxins were determined by correlating the peak area of the samples with the standard curves obtained by HPLC analysis of the standard solutions.

### 4.5. Determination of Endotoxins Concentrations by ELISA Assay 

The endotoxin concentrations in total mixed rations and different types of ensiled samples were determined by an endotoxin detection assay based on the ELISA technology. The EndoLISA^®^ test kit (Hyglos GmbH, Bernried, Germany, ref 609033) was used for the analysis. Extraction and testing of endotoxins were performed according to the manufacturer’s recommendations. The analytical method was validated by the kit manufacturer using matrices of maize and grass silage, and hay and TMR samples. Whole silage samples were air-dried, ground to pass through a 1 mm sieve and homogenized. The extraction of the samples was carried out in distilled water, 100 µg samples were diluted with 400 µg endotoxin-free water and vortexed for 2 min. Fluorescence detection was determined using the multimode microplate reader (Bio-Tek Instruments, Inc. Synergy HT, Winooski, VT, USA) at an excitation filter of 380 nm/band and an emission filter of 440 nm/band. A calibration curve of the endotoxin dilution standards was plotted using a standard concentration and the percentage inhibition of the corresponding standard. For determination, the endotoxin concentration was automatically calculated from the calibration curves and obtained by plotting the absorbance intensity against the logarithm of the analytic concentration. The measured fluorescence detection was automatically converted to the endotoxin’s concentration units, EU/mL. The lower limit of quantification (LLOQ) was 0.05 EU/mL (according to the manufacturer’s guidelines).

### 4.6. Statistical Analysis

The obtained data were analyzed using descriptive statistics (SPSS version 20.0, IBM Corp., Armonk, NY, USA). The results of *Enterobacteriaceae* counts were log transformed to log_10_ CFU/g prior to statistical analysis. The results were expressed as the mean value and the standard error of mean (SEM). The mean value of the results was expressed in at least 3 measurements.

## Figures and Tables

**Table 1:** Guidelines for mycotoxins levels in cattle feed in the EU.

Mycotoxin	Products Intended for Animal Feed	Maximum Level or Guidance Value in (μg/kg) *
Aflatoxin B_1_	Feed materials with the exception of:	50
	— Groundnut, copra, palm-kernel, cotton seed, bassu, maize and products derived from the processing thereof	20
	Complete feedingstuffs for cattle with the exception of:	50
	— Dairy cattle	5
Deoxynivalenol	Feed materials (*)	
	— Maize by-products	12,000
	— Compound feed	5000
Zearalenone	Feed materials (*)	
	— Maize by-products	3000
	— Compound feed of calves, dairy cattle, sheep (including lamb) and goats (including kids)	500
T-2 toxin	— Compound feed	250

* Relative to a feedingstuff with a moisture content of 12%.

**Table 2:** pH values of TMR and different types of ensiled forage (*n* = 119).

Samples	pH Values		
	Range	Mean	SEM
Maize silage (*n* = 34)	3.21–5.60	3.78	0.40
Grass silage (*n* = 20)	3.50–5.26	4.26	0.39
Haylage (*n* = 14)	3.78–4.53	4.12	0.23
TMR (*n* = 51)	3.65–6.73	4.61	0.61

**Table 3:** The total count of *Enterobacteriaceae* (log_10_ colony forming units’/g fresh weight) in TMR and different types of ensiled forage (*n* = 119).

Samples	*Enterobacteriaceae* (log_10_ CFU/g)			
	No. of Positive Samples (%)	Range	Mean	SEM
Maize silage (*n* = 34)	27 (79.42)	3.00–4.80	3.93	0.48
Grass silage (*n* = 20)	16 (80.00)	3.00–4.65	3.86	0.47
Haylage (*n* = 14)	11 (78.58)	3.10–4.70	3.06	1.73
TMR (*n* = 51)	7 (13.73)	3.00–4.73	3.35	1.43

**Table 4:** Mycotoxin contamination of TMR and different types of ensiled forage (*n* = 119).

Samples	Mycotoxin	No. of Positive Samples (%)	Range (μg/kg)	Mean (μg/kg)	SEM
Maize silage (*n* = 34)	AFB1	16 (47.06)	0.67–8.00	3.15	2.30
	ZEA	22 (64.71)	60.00–700.00	505.00	214.89
	DON	14 (41.18)	100.00–750.00	362.50	216.34
	T-2 toxin	25 (73.56)	12.33–284.05	110.09	83.91
Grass silage (*n* = 20)	AFB_1_	6 (30.00)	2.00–7.00	5.50	2.28
	ZEA	6 (30.00)	130.00–330.00	286.67	78.15
	DON	12 (60.00)	100.00–500.00	321.25	165.17
	T-2 toxin	16 (80.00)	39.00–260.00	154.17	89.86
Haylage (*n* = 14)	AFB_1_	6 (42.86)	2.00–10.00	5.08	2.98
	ZEA	9 (64.29)	70.00–750.00	307.75	273.33
	DON	8 (57.15)	50.00–500.00	198.13	182.21
	T-2 toxin	10 (71.43)	21.70–260.81	96.15	81.85
TMR (*n* = 51)	AFB_1_	31 (60.78)	1.03–5.00	2.42	1.11
	ZEA	25 (49.02)	70.00–700.00	377.60	248.96
	DON	28 (54.91)	50.00–500.00	283.94	185.10
	T-2 toxin	15 (29.42)	15.71–246.74	106.05	76.01

**Table 5:** Endotoxin concentrations in TMR and different types of ensiled forage (*n* = 119).

Samples		Endotoxins		
	No. of Positive Samples (%)	Range (EU/mL)	Mean (EU/mL)	SEM
Maize silage (*n* = 34)	28 (82.36)	41.90–541.40	266.59	200.45
Grass silage (*n* = 20)	16 (80.00)	23.49–525.00	124.02	124.12
Haylage (*n* = 14)	12 (85.72)	49.31–557.81	193.76	140.27
TMR (*n* = 51)	44 (86.28)	33.78–678.13	293.44	196.43

**Table 6:** HPLC analysis parameters for the mycotoxin detection.

Parameters	Mycotoxins			
	AFB1	DON	T-2	ZEA
Column temperature	30 °C	30 °C	40 °C	30 °C
Mobile phase	H_2_O/ACN/MeOH (60:20:30)	H_2_O/ACN/MeOH (94:3:3)	H_2_O/ACN (40:60)	H_2_O/ACN/MeOH (46:46:8)
Fluorescent detector, wavelength λ (nm) (excitation and emission)	365 and 435	-	381 and 470	274 and 418
UV detector λ (nm)	-	218	-	-
Flow rate (mL/min)	1	1	1	1
Injection volume (μL)	100	100	100	100
Limit of detection (LOD), μg/kg	0.2	20	1.4	3

UV—ultraviolet rays; H_2_O—water; ACN—acetonitrile; MeOH—methanol.

## Data Availability

The data presented in this study are available within the article.
